# Hybrid modelling using simulation and machine learning in healthcare

**DOI:** 10.1016/j.cor.2025.107278

**Published:** 2026-01

**Authors:** Ali Ahmadi, Masoud Fakhimi, Carin Magnusson

**Affiliations:** a Surrey Business School, University of Surrey, Guildford, UK; b School of Health Science, University of Surrey, Guildford, UK

**Keywords:** Modelling and simulation, Machine learning, Hybrid modelling, Healthcare, Data science

## Abstract

Modelling & Simulation (M&S) and Machine Learning (ML) methodologies have undergone significant advancements, enabling transformative applications across various industries. The integration of M&S and ML into a Hybrid M&S-ML approach leverages the unique strengths of both fields, offering enhanced model precision, improved efficiency, and more effective decision support. This review explores the increasing convergence of ML algorithms with traditional M&S methods- namely Agent-Based Modelling & Simulation, Discrete Event Simulation, and System Dynamics- in healthcare applications. Through a systematic review of 90 relevant studies, this article provides a comprehensive synthesis of the current state-of-the-art Hybrid M&S-ML in healthcare. Specifically, it examines the M&S and ML methodologies employed, associated software tools and programming languages, analyses integration patterns and data exchange mechanisms, and explores application domains, as well as the types and motivations for hybridisation. Key findings highlight prominent methodological and technical trends, as well as opportunities for combining M&S with ML to address healthcare challenges. These insights provide direction for modellers and data scientists in developing hybrid M&S–ML approaches that more effectively combine simulation capabilities with data-driven learning. The review also demonstrates the potential of such approaches to advance methodological innovation and support evidence-based decision-making in diverse healthcare contexts.

## 1 Introduction

The healthcare sector is widely recognised as a critical, complex and rapidly evolving industry that generates large volumes of data due to patient record-keeping, regulatory compliance, and clinical documentation (Salazar-Reyna et al., 2022). This data-intensive nature presents significant challenges in data management, processing, and extracting actionable insights to support decision-making (Raghupathi & Raghupathi, 2014). Despite the challenges, such as data privacy concerns and incomplete data entries that limit access to comprehensive datasets, the application of data-driven techniques in healthcare is increasingly seen as an opportunity to enhance patient care and outcomes, improve provider relationships with patients, advance personalised care, reduce healthcare expenses, and optimise healthcare operations (Pastorinoet al., 2019, Taipaluset al., 2022, Wang and Alexander, 2019). To sustain these improvements and drive further cost-effectiveness, continuous efforts to optimise healthcare systems remain essential (AbuKhousaet al., 2014, Basileet al., 2024).

Operational Research (OR) has played a critical role in advancing healthcare research, offering rigorous methodologies to support the design, evaluation, and optimisation of complex service delivery system challenges (Carter & Busby, 2023). Among OR methodologies, Modelling and Simulation (M&S) has proven to be an effective method for analysing system performance, supporting evidence-based decision-making, and informing long-term strategic planning (Hosteins, 2024, Klein and Thielen, 2024). Among M&S methodologies, Discrete Event Simulation (DES), System Dynamics (SD), and Agent-Based Modelling & Simulation (ABM&S) have been most widely adopted in healthcare (Karet al., 2024, Kovalchuket al., 2018, Palmeret al., 2018).

As healthcare systems grow increasingly complex, traditional single-method M&S approaches often struggle to fully capture the dynamic, adaptive, and interdependent nature of real-world processes (dos Santos et al., 2020). To overcome these limitations, researchers have explored the integration of multiple simulation approaches (e.g., DES-SD)—known as Hybrid Simulation (HS)—to achieve more comprehensive and realistic system representations (Harperet al., 2021, Mustafee et al., 2018). By leveraging the complementary strengths of each simulation technique, HS enhances the understanding of healthcare processes, improving predictive accuracy and decision support (Brailsfordet al., 2019, Pennyet al., 2023).

Despite their potential, HS is not without constraints. These include the need for simplifying assumptions to enable integration, increased computational demands, and persistent challenges in aligning models with real-world data (Gu & Kunc, 2020). To address these challenges and extend the applicability of simulation, researchers have increasingly turned to the integration of simulation with methods from other disciplines, leading to the growing adoption of hybrid modelling in OR and management science (Fakhimi & Mustafee, 2024). As Mustafee et al. (2018) highlight, the term hybrid modelling has gained traction in the literature to describe studies that combine simulation approaches—such as DES, SD, and ABM&S—with methodologies from the broader OR/MS domain or other disciplines, including the social sciences, computer science, and engineering. This interdisciplinary integration enhances model flexibility, improves adaptability, and comprehensively represents complex real-world systems (Tolk et al., 2021).

One of the key advancements in hybrid modelling is the integration of M&S with a data science approach, specifically machine learning (ML), enabling simulation models to leverage real-time analytics and statistical learning, while allowing ML to benefit from simulation-generated data, structured domain knowledge, and controlled experimentation. von Rueden et al. (2020) describe this complementarity by framing ML as a bottom-up, inductive approach derived from data and simulation as a top-down, deductive approach grounded in knowledge. Feldkamp (2024), however, points out that the boundary between the two is less clear in practice, as simulation can draw on data-driven methods at various stages, while ML often relies on simulation outputs or models, for example, in reinforcement learning or digital twin settings. These insights frame our examination of how Hybrid M&S–ML is being advanced in healthcare.

As healthcare systems become increasingly digitalised and data-rich, this integration is likely to create new opportunities for both research and practice. It broadens the scope and impact of M&S and ML in addressing healthcare challenges.

ML is a dynamic field of computational algorithms designed to mimic human cognitive processes by acquiring and processing data from the surrounding environment (Rebala et al., 2019). Advances in computational power and algorithm design have positioned ML as a critical enabler of intelligent systems capable of supporting complex decision-making in healthcare, generating predictive insights, and improving clinical workflows (Johnson, 2022). Its use has contributed to more targeted, adaptive, and efficient healthcare solutions (Awrahman et al., 2022).

M&S and ML share a common analytical foundation, with both fields leveraging mathematical modelling, optimisation, and statistical techniques to support complex decision-making (Hazen et al., 2018). Within the field of OR, there is a long-standing tradition of integrating methods from other disciplines to strengthen model development and application (Ranyard et al., 2015). Ormerod (2020) argues that expanding the OR toolkit fosters new research opportunities, while Pruyt (2016) highlights a major shift from traditional data-poor modelling to data-rich environments. This shift has created new opportunities for combining ML with M&S, particularly to take advantage of large-scale data, improve model precision, and support automation and scalability. This hybrid approach is particularly valuable in healthcare, where complex, data-intensive problems demand both robust simulation techniques and AI-driven adaptability.

Recent studies have shown increasing interest in combining M&S and ML within healthcare contexts (e.g., Ala et al., 2022; Botzet al., 2022, Greasley and Edwards, 2021). These studies explore the potential of linking system-oriented, dynamic simulation techniques with data-driven ML approaches to better represent, analyse, and manage healthcare systems. This reflects a broader trend in healthcare analytics towards integrated, computationally sophisticated methods that can support more scalable and adaptive decision-making.

Given the increasing adoption of Hybrid M&S–ML approaches and their potential to bridge complementary analytical capabilities, a systematic review is needed to consolidate current knowledge and identify future research directions. Existing literature reviews tend to examine M&S or ML in isolation. For example, dos Santos et al. (2020) and Kar et al. (2024) focus on HS in healthcare but do not consider its integration with ML. Conversely, reviews by Salazar-Reyna et al. (2022) and Subrahmanya et al. (2022) examine ML applications in healthcare without considering M&S. Within the Hybrid M&S–ML literature, systematic reviews are scarce; the few that exist have primarily been conducted in the context of supply chain management (Badakhshanet al., 2024, Kogler and Maxera, 2025). The only review identified that considers both simulation and AI in the context of healthcare is by Ponsiglione et al. (2024), which focuses specifically on healthcare management challenges and is based on a dataset of 11 papers published between 2012 and 2021. Therefore, a comprehensive review of Hybrid M&S-ML applications in healthcare is needed to explore their synergies, methodological developments, and potential to address complex healthcare challenges. Table 1 provides a summary of the literature review studies.

**Table 1:** Review of the literature review studies.

**Authors**	**Domain Focus**	**Time Frame**	**Topic Focus**	**Key Contribution**
Brailsford et al. (2019)	Operational Research	2000–2016	Hybrid Simulation	Reviews hybrid simulation in OR; no integration with ML.
dos Santos et al. (2020)	Healthcare	2012–2018	Hybrid Simulation	Reviews hybrid simulation in healthcare; no integration with ML.
Salazar-Reyna et al. (2022)	Healthcare engineering systems	2004–2019	Data Science	Reviews data science applications in healthcare; no integration with M&S.
Subrahmanya et al. (2022)	Healthcare	2010–2020	Data Science	Reviews data science applications in healthcare; no integration with M&S.
Kar et al. (2024)	Healthcare	2008–2023	Hybrid Simulation	Reviews hybrid simulation in healthcare; no integration with ML.
Ponsiglione et al. (2024)	Healthcare	2013–2021	Hybrid M&S–AI	Reviews hybrid M&S–AI with a focus on healthcare management challenges.
Armenia et al. (2024)	Supply chain management	2000–2025	Hybrid M&S–ML	Reviews hybrid M&S–ML in supply chain management.
Kogler and Maxera (2025)	Supply chain and logistics	2013–2025	DES/ABM&S–ML	Reviews hybrid M&S–ML in supply chains and logistics.
This Study	Healthcare	Until October 2024	Hybrid M&S–ML	First systematic review of hybrid M&S–ML in healthcare, addressing roles, methods, data flow, and integration approaches.

This review aims to answer five key research questions:

o RQ1: What healthcare domains are most frequently targeted in hybrid M&S–ML studies, and how do these application areas relate to the nature of studies conducted?
o RQ2: Which M&S techniques, ML tasks, algorithms, and integration approaches are most prevalent in hybrid M&S–ML studies, and what patterns emerge in their application across different implementation levels?
o RQ3: What are the stated aims and functional roles of ML within hybrid M&S–ML models in healthcare studies?
o RQ4: How are ML methods deployed across the stages of the M&S lifecycle, and what integration setups and data flow mechanisms are used to facilitate this deployment?
o RQ5: How are hybrid M&S–ML models in healthcare validated, verified, and implemented across different study types and maturity levels?

The remainder of the paper is structured as follows: Section 2 outlines the search methodology and review framework. Section 3 presents the results of the literature analysis. Section 4 discusses the key insights and takeaways from the results. Finally, Section 5 concludes the paper with recommendations for future research.

## 2 Methodology

### 2.1 Review protocol

The review followed a structured protocol to identify, select, and analyse relevant studies involving the integration of M&S and ML in healthcare. The Web of Science (WOS) database was chosen as the primary source due to its comprehensive coverage of high-quality, peer-reviewed literature across multiple disciplines. Given the interdisciplinary nature of the subject, a structured topic search was conducted using three main themes: M&S, ML, and healthcare applications. The *first theme* of keywords focused on M&S, covering the three most widely used simulation approaches —namely, DES, SD, and ABM&S, as well as their possible combinations (Hybrid Simulation, e.g., SD-DES). The *second theme* captured a range of ML-related terms, and the *third theme* filtered for healthcare-specific studies. To ensure quality and relevance, the search was limited to English-language, peer-reviewed articles published up to 30th October 2024. The complete list of keywords for each theme is provided in Table 2.

**Table 2:** The review protocol.

**Review Conditions**	**Description**	
**Use of Database**	Web of Science. Comprehensive coverage of high-quality journals and conferences, providing access to bibliographic information for ∼8,500 impact-factor journals.	
**Search Strings**	Category	Search Terms
	Modelling and Simulation	(“Hybrid Simulation”) **OR** (“DES” **AND** “System dynamics”) **OR** (“DES” **AND** “Simulation”) **OR** (”ABS“ **AND** ”Simulation“) **OR** (”DES“ **AND** ”ABS“) **OR** (”ABS“ **AND** ”System Dynamic*”) **OR** (“SD” **AND** “Simulation”) **OR** (“System Dynamic*”) **OR** (“Discrete event*”) **OR** (“Agent based*”) **OR** (“multi agent*” **AND** “Simulation”)
	Machine Learning	(“Machine Learning”) **OR** (“Deep Learning”) **OR** (“Artificial Intelligence”) **OR** (“AI”) **OR** (“big data”) **OR** (“data mining”) **OR** (“Neural Network”) **OR** (“Decision Tree”) **OR** (“Support Vector Machine”) **OR** (RNN) **OR** (CNN) **OR** (SVM) **OR** (“Random Forest”) **OR** (“Data Scienc*”)
	Healthcare	(“Health*” **OR** “diseas*” **OR** “Care” **OR** “Medic*” **OR** “Clinic*” **OR** “Illn*” **OR** “Dent*” **OR** “Pharma*” **OR** “patient*” **OR** “Surge*”)
**Quality Control and Article Suitability Review**	**Inclusion Criteria**: 1. Articles published up to the end of October 2024. 2. Peer-reviewed articles, including research proposals. **Exclusion Criteria**: 1. Grey literature, trade publications, and editorials. 2. Non-English articles.	

**Fig. 1:**
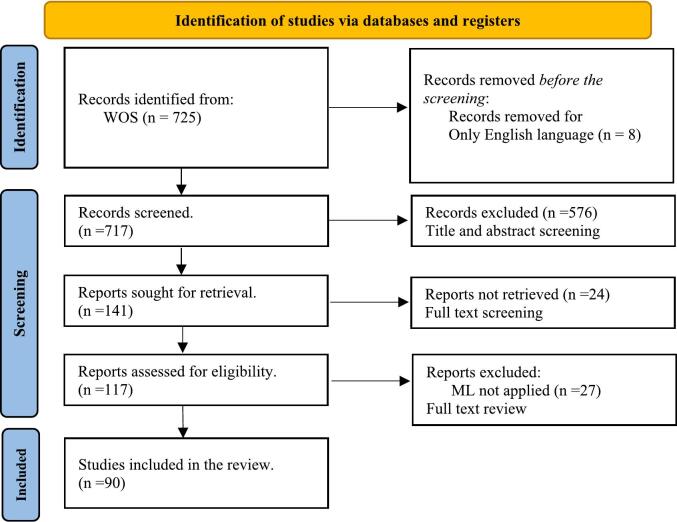
PRISMA 2020 flow diagram of the study selection process Haddaway et al. (2022).

The literature selection process adhered to the Preferred Reporting Items for Systematic Reviews and Meta-Analyses (PRISMA) guidelines, ensuring a structured and transparent approach (Pageet al., 2021, Pageet al., 2021). The initial database search used the predefined keywords to retrieve 725 articles. After applying the exclusion criteria, 717 articles remained. A title and abstract screening phase were then conducted, removing 576 articles that did not meet the scope of Hybrid M&S–ML in healthcare. The remaining 141 articles were subjected to full-text screening. This stage focused on identifying whether each study explicitly combined M&S and ML within healthcare applications. This resulted in a shortlist of 117 articles. A final round of in-depth review excluded 27 additional articles that did not fully meet the inclusion criteria, leaving a total of 90 studies for systematic analysis. The review process was undertaken collaboratively by the three authors. Initial screening and categorisation were performed by one author, with the other two authors providing oversight. Consistency checks were conducted by comparing classifications, and any uncertainties were discussed collectively until consensus was reached. Fig. 1 presents the PRISMA flow diagram detailing the review process.

### 2.2 Review framework

To guide the classification and analysis of the selected literature, this study adopts the Profiling, Problems, Models, and Outcomes (PPMO) framework, a structured approach that has been widely used in previous simulation reviews (e.g., Alidoostet al., 2025, Karet al., 2024, Mustafeeet al., 2021). The PPMO framework offers a systematic structure for organising and synthesising diverse literature based on key dimensions of research design and contribution.

In this review, the framework was extended to suit the hybrid modelling literature, enabling more effective capture of technical and methodological features, and enhancing its capacity to reflect the varied roles ML plays within M&S research. By introducing more granular variables, the modified framework facilitated a deeper and more systematic assessment of the interactions between M&S and ML, moving beyond the traditional focus of M&S-only literature reviews. The revised framework was also aligned with the review’s research questions, ensuring a comprehensive and structured synthesis of the field.

The framework comprised four components. The first, *Profiling Research*, captures publication characteristics and disciplinary focus. The second, *Problem Definition and Context*, identified the healthcare domain and the type of studies. The third, *Model Development and Hybridisation*, focused on the methodological configuration of the hybrid M&S–ML models, detailing the simulation methods, ML tasks and algorithms, programming languages, software tools, and the integration strategies employed. It also examined how data is exchanged between M&S and ML components, including the formats and intermediary mechanisms used. The final component, *Study Outcomes*, assessed the progression of studies from conceptual development to application, with attention to the reported level of implementation and the nature of verification and validation practices. The complete structure of the review framework is presented in Table 3.

**Table 3:** Review framework.

Profiling Research (P) (Section 2.3)	Problem Definition and Context (P) (Section 3.1)
• Publication Characteristics • Publication Discipline	• Context of Application • Type of Study

Model Development and Hybridisation (M) (Section 3.2)	Study Outcome (O) (Section 3.3)

• M&S Technique and Software • ML Tasks and Aim of Use with M&S • ML Categories and Algorithms • Programming Language • Integration of M&S with ML o Roles of ML Across the M&S Process o M&S-ML Integration Setup o Data Flow Mechanism o Integration Method	• Level of Implementation • Verification & Validation

### 2.3 Profiling research

This section examined the publication characteristics and disciplinary diversity of the selected studies to identify trends in the development of Hybrid M&S–ML research in healthcare. Key variables include publication year and type, which help trace the field’s growth and shifts in research emphasis over time. The analysis also considered the studies' disciplinary affiliations to highlight the interdisciplinary nature of this research area and to identify which domains are most actively contributing to the integration of M&S and ML in healthcare contexts.

#### 2.3.1 Publication characteristics

The dataset for this review comprises 73 journal articles and 17 conference proceedings papers. In contrast, Kar et al. (2024) reported that over 60 % of HS studies in healthcare were published in conference proceedings. This difference may reflect the multidisciplinary nature of Hybrid M&S–ML, where journal outlets are often prioritised for methodological depth and theoretical contribution.

Among the 90 journal and conference papers, a small number of journals and conferences appeared more than once in the dataset. These included the Journal of the Operational Research Society, PLOS Computational Biology, and the Journal of Medical Systems, each contributing three articles.

**Fig. 2:**
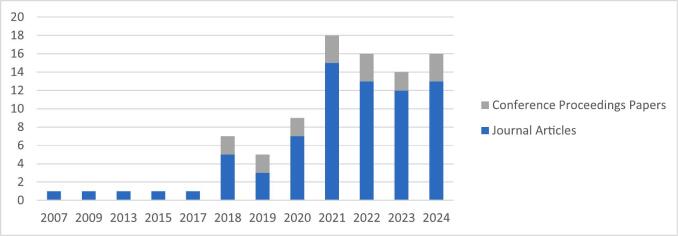
Publication Trend of Hybrid M&S–ML Studies in Healthcare.

As illustrated in Fig. 2, Hybrid M&S–ML uptake in healthcare research has shown a clear upward trend since 2018. This growth reflects not only the inherent complexity and data-rich nature of healthcare systems—which has driven interest in decision support approaches combining predictive modelling with adaptive analytics—but also two broader developments: the increasing adoption of ML methods within the OR community over the past decade (Talavera & Luna, 2020) and the continued prominence of healthcare as a core application area for M&S and HS (dos Santos et al., 2020).

#### 2.3.2 Publication discipline

The WOS assigns categories based on several criteria, including subject matter, journal scope, author and editorial affiliation, funding agencies providing grant support, and cited references, which are compared to the scope notes of their respective categories (Clarivate, 2025). This section analysed the disciplinary composition of the reviewed studies based on their WOS subject categories. The aim is to understand which academic disciplines are contributing to Hybrid M&S–ML research in healthcare and to assess the extent of interdisciplinary engagement in this domain.

Based on the WOS classifications of the 90 selected articles, comprising 164 subject category entries, related fields were aggregated into broader groups through a qualitative mapping process. Four primary disciplinary clusters were identified: 1) *Computer Science and Engineering*, 2) *Medical and Healthcare*, 3) *Operational Research and Management Science (ORMS) and Business*, and 4) *Others (*e.g.*, Environmental Science, Mathematics)*. For instance, Medical Informatics and Health Care Sciences & Services were grouped under *Medical and Healthcare*, while various Computer Science subfields and general Engineering were grouped under *Computer Science and Engineering*. Articles could fall into multiple categories based on their thematic scope.

The *Computer Science and Engineering* cluster accounted for the largest share, with 70 instances and 12 research areas, followed by *Medical and Healthcare* with 62 instances, and *ORMS and Business* with 19 instances. The remaining 13 instances came from categories such as Mathematics and Multidisciplinary Sciences. Given the multidisciplinary nature of the field, this distribution underscores the leading role of computational techniques in Hybrid M&S-ML research, while also highlighting the strong application focus from the healthcare domain.

**Fig. 3:**
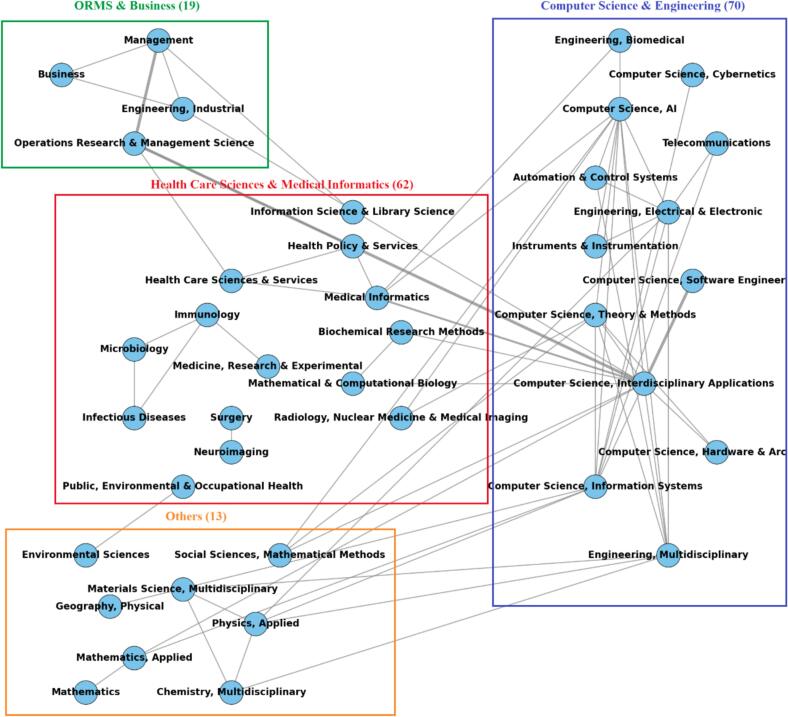
Interdisciplinary Structure of Hybrid M&S–ML Research in Healthcare.

The relatively lower representation of *ORMS and business* may reflect funding priorities and cross-disciplinary challenges, with the growing focus on AI and ML in healthcare further contributing to the underrepresentation of ORMS. The data reveals limited connections between ORMS and healthcare, which is interesting given that M&S, a core component of Hybrid M&S-ML, is one of the most widely applied OR methods in healthcare. The co-occurrence diagram in Fig. 3 illustrates the interconnections among disciplines, highlighting the interdisciplinary nature of the research and the frequency with which these categories co-occur in hybrid M&S–ML healthcare studies.

## 3 Findings

This section presents the review's findings, structured according to the proposed extended PPMO framework provided in Section 2. Each part of the analysis directly corresponds to the review's research questions. Section 3.1 addresses RQ1 by classifying healthcare domains and problems studied. Section 3.2 responds to RQ2-RQ4, examining the integration of M&S and ML, including the M&S techniques, ML tasks, and integration methods used in the reviewed studies. Section 3.3 answers RQ5, evaluating the levels of implementation and the verification and validation processes in the studies.

### 3.1 Problem definition and context (P)

This section examines the healthcare domains where Hybrid M&S–ML has been applied, highlighting the practical focus and problem areas addressed. It also classifies the studies based on their research orientation to distinguish between application-led work, conceptual frameworks, and theoretical or methodological contributions.

#### 3.1.1 Context of application

This analysis categorises the reviewed studies based on their primary healthcare applications, identifying key thematic areas within the literature. The literature was systematically reviewed, and each paper was initially categorised based on its primary healthcare application. A comparative analysis identified conceptual similarities, leading to the merging of related categories through iterative refinement. This process resulted in the consolidation of categories into four main themes (see Table 4): Hospital Operations and Resource Management, Infectious Disease Modelling and Public Health Interventions, Clinical Decision Support and Treatment Pathway, and Biological and Physiological Modelling.

**Table 4:** Distribution of reviewed studies across key healthcare application themes.

**Application Context**	**Number of Articles**
*Hospital Operations and Resource Management*	37
*Infectious Disease Modelling and Public Health Interventions*	33
*Clinical Decision Support and Treatment Pathway*	15
*Biological and Physiological Modelling*	5

Thirty-seven articles (41 %) within the dataset concerned various aspects of hospital operations and resource management, including resource allocation, performance improvement and patient flow optimisation. For example, Shokouh et al. (2022) combined DES, Artificial Neural Network (ANN), and Genetic Algorithm (GA) to enhance emergency department (ED) efficiency by reducing patient waiting times and improving resource utilisation. This study demonstrated how strategic resource allocation—such as adjusting triage staff and physician availability—can significantly improve ED performance. Similarly, Uriarte et al. (2017) combined DES with data mining to optimise ED decision-making and resource use, improving key performance indicators such as time to physician consultation and patient throughput.

Thirty-three articles (37 %) focused on infectious disease modelling and public health interventions, including the modelling of disease transmission and the evaluation of non-pharmaceutical interventions (NPIs). For example, Al-Bazi et al. (2024) employed a hybrid ABM&S and decision tree (DT) model to study the effect of ventilation systems on COVID-19 exposure. Lutz and Giabbanelli (2022) explored a similar hybrid ABM&S-DT to predict future COVID-19 trajectories, illustrating how ML *meta*-models can enable faster decision-making in evolving health crises.

Fifteen articles (17 %) were centred on clinical decision support and treatment pathways, including patient-level modelling, treatment response, and personalised care. For example, Shojaati and Osgood (2023) combined HS and Bayesian network (BN) to evaluate medication effectiveness, showing that reducing opioid prescriptions could significantly lower medical and non-medical overdose rates over five years. Kumar et al. (2021) used ABM&S with k-Nearest Neighbour (kNN) to model the effects of mindfulness programmes on emotional regulation and heart rate variability, proposing personalised care systems that adapt to individual patient responses.

Five studies (5 %) focused on biological and physiological modelling, including applications in disease diagnosis, immune response simulation, and cellular and within-body dynamics. Schneckenreither et al. (2021) introduced ABM&S-ANN to simulate macroscopic pigmentation patterns for cancer diagnosis, linking underlying cellular behaviour to dermatoscopic imaging. Cockrell and An (2021) applied GA to calibrate an ABM&S of acute systemic inflammation, maintaining biological heterogeneity observed in clinical data and enhancing model realism.

The predominant focus remains on hospital operations and resource management, with infectious disease modelling, particularly related to COVID-19, making up 85 % of the studies published after 2020. Section 3.1.2 further categorises these studies by study type.

#### 3.1.2 Type of study

This analysis adopts the classification scheme proposed by Brailsford et al. (2019), which categorises studies into three types: Type A—application-based studies or case studies; Type B—conceptual frameworks supported by illustrative applications; and Type C—theoretical, conceptual, or methodological contributions. Among the 90 studies included in this systematic review, the majority fall into either Type A (42 studies, 47 %) or Type B (40 studies, 44 %), with a smaller number classified as Type C (8 studies, 9 %).

Type A studies primarily focused on hospital operations and resource management (17 studies, 40 %) and infectious disease modelling and public health interventions (17 studies, 40 %). In these studies, ML was commonly used for forecasting admissions (e.g., Gartner and Padman, 2020, Oziket al., 2021, Snyder and Smucker, 2022), optimising emergency resources (e.g., Fairleyet al., 2019, Kimet al., 2021; Theeuwes et al., 2021), or predicting outbreak trends (e.g., Chenet al., 2019, Krivorotkoet al., 2023, Olave-Rojas and Nickel, 2021). It is followed by 15 % on clinical decision support and treatment pathways (15 %) (e.g., Cess and Finley, 2023, Nanet al., 2024), and biological and physiological modelling (5 %) (e.g., Monostoriet al., 2021, Schneckenreitheret al., 2021).

Type B studies shared a similar distribution across application areas (42 %, 35 %, 15 %, and 8 %, respectively). These included hybrid DES–ML models for hospital capacity planning (e.g., Ortiz-Barrioset al., 2023, Ortiz-Barrioset al., 2024) and ML-enhanced SEIR models for epidemic control (e.g., Abdulkareemet al., 2020, Snellmanet al., 2024, Valtchevet al., 2021). Type C contributions were limited, consisting of one review (Siettos & Russo, 2013) and a few conceptual studies (e.g., Calvaresiet al., 2020, Gehlotet al., 2022).

**Fig. 4:**
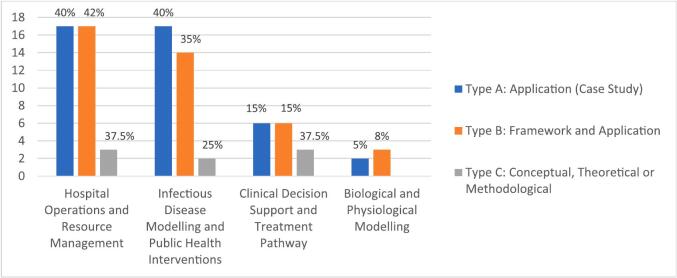
Type of Study (Adopted from Brailsford et al. 2019) *(Percentages are calculated relative to the total number of studies within each type.)*.

Fig. 4 presents the distribution of papers across the three study types, mapped against the application contexts introduced in Section 3.1.2.

We also examined the geographical distribution of case studies reported in Type A and Type B papers. Of the 90 studies, 63 provided identifiable location data. The United States was the most frequently represented (17 studies), followed by Canada, Iran, and Russia (4 studies each), and Germany, Ghana, Italy, and Türkiye (3 studies each). Brazil, China, South Korea, and South Africa each featured in two studies. Single case studies were reported from a wide range of countries, including Australia, Austria, Bangladesh, the European Union, France, Israel, Jordan, the Netherlands, Saudi Arabia, Spain, Sweden, Taiwan, the United Kingdom, and one cross-national study involving both the United States and China.

### 3.2 Model development and hybridisation (M)

This section provides an in-depth examination of the M&S and ML methodologies, algorithms, languages, and tools employed in the reviewed studies. It also explores the motivations for integrating M&S with ML, the methods of combination, the data formats used, and the techniques applied for model integration in healthcare contexts.

#### 3.2.1 M&S technique and software

Among the reviewed studies, ABM&S emerged as the most widely adopted M&S technique integrated with ML, featured in 56 articles (62 %). DES followed, appearing in 29 studies (32 %), while SD was used in only one instance (1 %). Additionally, four studies (5 %) employed HS methods in conjunction with ML (i.e., ABM&S-DES-ML or ABM&S-Monte-Carlo Simulation-ML).

**Fig. 5:**
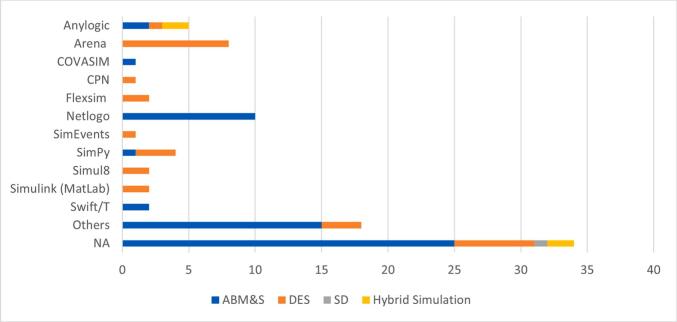
M&S Techniques and Corresponding Software Platforms in Hybrid M&S–ML Healthcare Studies.

Of the 90 studies reviewed, the simulation software used was identifiable in 62 cases—either explicitly reported or inferred from the study description. Twenty-four studies used commercial tools, while 38 relied on open-source platforms. Among the open-source implementations, NetLogo was the most common (10 articles), followed by SimPy (4 articles) and Swift/T (2 articles). A further 14 studies used other open-source tools, including Java-based simulation platforms. On the commercial side, Arena appeared in 8 articles, making it the most frequently used proprietary tool, followed by AnyLogic (5 articles). Other tools included Simulink, Simul8, and FlexSim, each used in two studies, along with four studies using other commercial platforms. Fig. 5 provides an overview of the distribution of M&S approaches alongside the software tools employed in Hybrid M&S–ML studies in healthcare. In 34 studies, the software was not disclosed or could not be determined and was therefore recorded as NA.

#### 3.2.2 ML tasks and aim of use with M&S

This section explores the specific purposes for which ML was integrated with M&S across the reviewed studies. To guide the analysis, we employed the well-established classification of ML tasks into predictive, prescriptive, and descriptive categories. This typology provides a structured basis for interpreting the functional role ML plays in hybrid models, clarifying whether it is used to forecast outcomes, support decision-making, or extract insights from complex data.

Among the 90 studies, predictive analytics emerged as the most common aim of ML integration, reported in 58 studies (64 %). These studies predominantly used ML to anticipate future states, model patient outcomes, or forecast system performance. Of these, 33 studies (57 %) applied regression-based or time-series forecasting techniques (e.g., Krivorotkoet al., 2023, Ricciardiet al., 2022, Tang, 2024). A further 11 studies (19 %) applied classification algorithms (e.g., Calvaresiet al., 2020, Gradonet al., 2021, Harrisonet al., 2023), while 14 studies (24 %) used ML for broader predictive tasks, such as estimating unknown variables or generating scenario-driven forecasts to support simulation (e.g., Larieet al., 2021, Mohammadzadeh and Safdari, 2015).

Prescriptive analytics was reported in 27 studies (30 %), where ML was used to guide decision-making, optimise resource allocation, or support policy evaluation. Within this group, 17 studies combined ML with optimisation models (e.g., Alimet al., 2024, Forbus and Berleant, 2023, Whiteet al., 2022), while seven studies used reinforcement learning approaches to enable adaptive decisions in dynamic contexts (e.g., Cockrellet al., 2022, Guoet al., 2022). Two studies (Cockrell et al. (2021) and Abdulkareem et al. (2019)) deployed ML heuristics to guide model selection or inform the evaluation of policy alternatives. One study (Abdulkareem et al., 2020) applied recommender systems for adaptive risk response in socio-environmental systems.

**Fig. 6:**
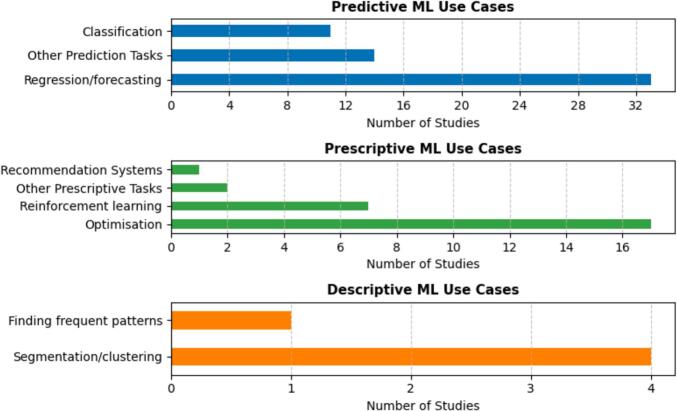
Distribution of ML Task in Hybrid M&S-ML Healthcare Studies.

Descriptive analytics appeared in only five studies (6 %), where ML was used to extract latent structures or uncover patterns in healthcare data. Four of these studies applied clustering techniques (e.g., Ceglowskiet al., 2007, Uriarteet al., 2017), while one study used frequent pattern mining with M&S (e.g., Dianov et al., 2022). Fig. 6 depicts the distribution of studies based on the primary purpose of ML integration with M&S, categorised as predictive, prescriptive, or descriptive tasks.

#### 3.2.3 ML categories and algorithms

This section examines the categories of ML applied—namely, supervised, unsupervised, and semi-supervised learning—as well as the final algorithms selected for integration with simulation models. Given that many studies tested multiple ML techniques, this review focuses on the algorithm ultimately implemented in the hybrid model, typically selected based on performance criteria such as accuracy or predictive reliability. In addition to reviewing algorithm frequency, this section also maps ML techniques to the simulation paradigms with which they were paired, providing a structured overview of how ML was operationalised within DES, ABM&S, SD, and hybrid simulation frameworks.

Among the reviewed studies, supervised learning emerged as the dominant ML category, reported in 63 studies. Unsupervised learning appeared in 15 studies, while three studies employed semi-supervised learning.

In terms of specific algorithms, Artificial Neural Networks (ANN) were the most frequently used, featured in 24 studies (e.g., Chopraet al., 2023, Cockrell and Axelrod, 2023, Huet al., 2022). Random Forest (RF) followed, appearing in 10 studies (e.g., Ortiz-Barrioset al., 2023, Ortiz-Barrioset al., 2024, Stolfi and Castiglione, 2021). Other algorithms included Support Vector Machines (SVM), Gradient Boosting Machines (GBM), k-Nearest Neighbour (kNN), and clustering-based methods. While many studies reported the testing of multiple ML methods, the algorithm reviewed here corresponds to the one ultimately selected for integration with the simulation model—typically justified based on predictive accuracy, robustness, or interpretability.

To support a more nuanced understanding, this review classified the algorithm used by the simulation paradigm. Table 5, Table 6, Table 7, Table 8 provide a breakdown of ML tasks, aims, learning categories, and algorithm selection in studies involving ABM&S, DES, SD, and HS.

##### 3.2.3.1 Hybrid ABM&S-ML

In ABM&S–ML studies (Table 5), ***predictive tasks*** were the most prevalent, appearing in 35 of 56 studies (63 %). Eighteen of these focused on regression or forecasting tasks and employed diverse ML techniques spanning supervised, unsupervised, and semi-supervised approaches. Commonly used algorithms included ANN (six studies), GBM (three), and RF (three) (e.g., Chopraet al., 2023, Chiuet al., 2024, Hatnaet al., 2024). Classification tasks, reported in seven studies, used techniques such as SVM, KNN, hierarchical clustering, decision trees, linear discriminant analysis, and ANN to model agent behaviour or state transitions (e.g., Beermanet al., 2023, Alexanderet al., 2019). An additional ten studies addressed other predictive tasks using hybrid or less conventional algorithms (e.g., Abdulkareem et al., 2018), often for estimating unknown inputs or simulating complex behavioural dynamics.

**Table 5:** ML tasks and algorithms in ABM&S–ML studies.

ML Task	Use Case	ML category	Main ML algorithms used	Studies
Predictive (35)	Regression /forecasting (18)	Supervised Learning (14) Unsupervised Learning (3) Semi-Supervised Learning (1)	ANN (6), GBM (3), RF (3), BN (1), CDT (1), EA (1), LR (1), NA (1), LSTM (1)	Tang, 2024, Hatnaet al., 2024, Abu Sufianet al., 2024, Chiuet al., 2024, Chopraet al., 2023, Krivorotkoet al., 2023, Huet al., 2022, Lutz and Giabbanelli, 2022, Schneckenreitheret al., 2021, Barneset al., 2020, Laskowskiet al., 2009, Siettos and Russo, 2013, Salibaet al., 2023, Anirudhet al., 2022, Stolfi and Castiglione, 2021, Perumal and van Zyl, 2020, Stolfi and Castiglione, 2020, Chenet al., 2019
	Classification (7)	Supervised Learning (7)	ANN (1), HC (1), DT (1), KNN (1), LDA (1), SVM (1), NA (1)	Al-Baziet al., 2024, Gradonet al., 2021, Calvaresiet al., 2020, Alexanderet al., 2019, Harrisonet al., 2023, Beermanet al., 2023, Batataet al., 2018
	Other Prediction Tasks (10)	Supervised Learning (7) Unsupervised Learning (2) Semi-Supervised Learning (1)	ANN (3), BN (3), KNN (2), LSTM (1), SVM (1)	Cess and Finley, 2023, Cockrell and Axelrod, 2023, Jorgensenet al., 2022, Perumal and van Zyl, 2022, Kumar et al. (2021), Larieet al., 2021, Oziket al., 2021, Valtchevet al., 2021, Abdulkareemet al., 2018, Mohammadzadeh and Safdari, 2015
Prescriptive (19)	Optimisation (10)	Supervised Learning (5) Unsupervised Learning (5)	EA (4), ANN (2), KNN (1), RF (1), EL (1), AL (1)	Hoet al., 2023, Wanget al., 2024, Whiteet al., 2022, Sulis and Terna, 2021, Cockrell and An, 2021, Nardiniet al., 2021, Suliset al., 2020, Oziket al., 2018, Yousefiet al., 2018, Yousefi and Yousefi, 2020
	Reinforcement (6)	Reinforcement Learning (6)	RL (6)	Lazebnik, 2023, Cockrellet al., 2022, Guoet al., 2022, Monostoriet al., 2021, Khatami and Gopalappa, 2021, Shuvoet al., 2024
	Recommendation Systems (1)	Supervised Learning (1)	BN (1)	Abdulkareem et al. (2020)
	Other Prescriptive Tasks (2)	Supervised Learning (1) Semi-Supervised Learning (1)	BN (1), AL (1)	Cockrellet al., 2021, Abdulkareemet al., 2019
Descriptive (2)	Segmentation /clustering (1) Finding frequent patterns, associations, and correlations (1)	Supervised Learning (1) Unsupervised Learning (1)	SOM (1), ANN (1)	Snellmanet al., 2024, Dianovet al., 2022

***Prescriptive tasks*** were investigated in 19 studies (34 %), of which ten focused on optimisation (e.g., Nardiniet al., 2021, Suliset al., 2020, Yousefi and Yousefi, 2020). Six studies applied reinforcement learning (RL)—the highest concentration of RL use across all simulation paradigms. The remaining prescriptive studies implemented recommender systems or other ML-driven interventions to support adaptive responses (e.g., Dianovet al., 2022, Shuvoet al., 2024, Oziket al., 2018).

***Descriptive tasks*** were comparatively rare, reported in only two ABM&S–ML studies (3 %). These studies used unsupervised learning techniques such as self-organising maps or ANN to identify emergent patterns in simulated healthcare environments (e.g., Dianovet al., 2022, Snellmanet al., 2024).

##### 3.2.3.2 Hybrid DES-ML

As shown in Table 6, hybrid DES–ML studies were predominantly focused on ***predictive tasks***, which appeared in 18 of the 29 studies (62 %). Within this group, 13 studies employed regression and forecasting techniques using supervised learning algorithms such as RF and ANN, primarily to enhance input realism or to support data-driven parameterisation (e.g., Atalan et al., 2022, Medvedet al., 2018). A further four studies used ML to predict simulation outcomes based on varying system configurations, thereby supporting downstream analysis and scenario testing (e.g., Gul et al., 2020).

**Table 6:** ML tasks and algorithms in DES–ML studies.

ML Task	Use Case	ML category	Main ML algorithms used	Studies
Predictive (18)	Regression /forecasting (13)	Supervised Learning (12) NA (1)	RF (5), ANN (3), LR (1), GB (1), CART (1), MLM (1), NA (1)	Ortiz-Barrioset al. (2024), Ortiz-Barrioset al. (2024), Ortiz-Barrioset al., 2023, Wornowet al., 2023, Atalan et al. (2022), Snyder and Smucker, 2022, Kimet al., 2021, Ricciardiet al., 2022, Theeuwes et al. (2021), Timilehin and van Zyl, 2021, Medvedet al., 2018, Mesabbahet al., 2019, Gehlotet al., 2022
	Classification (1)	Supervised Learning (1)	DT (1)	Gartner and Padman (2020)
	Other Prediction Tasks (4)	Supervised Learning (4)	ANN (2), GB (1), Other (1),	Asgaryet al., 2021, Fairleyet al., 2019, Gulet al., 2020, Kovalchuket al., 2018
Prescriptive (8)	Optimisation (7)	Supervised Learning (6) NA (1)	ANN (4), GBM (1), GNB (1), NA (1)	Borestaet al., 2024, Alimet al., 2024, Forbus and Berleant, 2023, Abuhayet al., 2023, Ying and Lin, 2023, Shokouhet al., 2022, Rabbaniet al., 2018
	Reinforcement (1)	Reinforcement Learning (1)	RL (1)	Hijry (2024)
Descriptive (3)	Segmentation /clustering (3)	Unsupervised Learning (3)	K-Means (1), FPM (1), SOM (1)	Valiet al., 2022, Uriarteet al., 2017, Ceglowskiet al., 2007

***Prescriptive*** applications were reported in eight studies (28 %), where ML algorithms such as GBM, GNB, and ANN were integrated to support optimisation, decision-making, or adaptive policy evaluation (e.g., Abuhayet al., 2023, Borestaet al., 2024, Rabbaniet al., 2018). Reinforcement learning was employed in only one study (Hijry, 2024), indicating its relatively limited adoption in DES–ML contexts.

***Descriptive*** analytics were less common, appearing in three studies, which applied unsupervised methods such as clustering and segmentation to analyse healthcare system patterns or classify input profiles (e.g., Ceglowskiet al., 2007, Valiet al., 2022). Although DES is typically oriented towards process-centric analysis, these examples demonstrate the added value of integrating ML to support exploratory data analysis and pattern recognition within discrete event frameworks.

##### 3.2.3.3 Hybrid SD-ML

SD–ML integration was identified in only one study among the reviewed literature (Table 7). Liu and Pu (2022) applied supervised learning techniques—specifically, Multi-Layer Perceptron (MLP) and SVM—to perform a classification task within a System Dynamics framework. No studies were found that employed prescriptive or descriptive ML integration with SD models.

**Table 7:** ML tasks and algorithms in SD–ML studies.

ML Task	Use Case	ML category	Main ML algorithms used	Studies
Predictive (1)	Classification (1)	Supervised Learning (1)	ANN (1)	Liu and Pu (2022)

##### 3.2.3.4 HS-ML

A small subset of studies explored hybrid simulation (HS) models integrated with ML, as summarised in Table 8. Two studies combined ABM&S and DES to support predictive tasks such as classification and forecasting, with ML used to improve input quality and support more realistic system representations (e.g., Kim, 2024, Olave-Rojas and Nickel, 2021). These hybrid configurations enabled the modelling of both individual-level behaviours and system-level processes, providing a richer representation of healthcare dynamics.

**Table 8:** ML tasks and algorithms in HS–ML studies.

Methods	ML Task	Use Case	ML category	Main ML algorithms used	Studies
ABM&S-DES (2)	Predictive (2)	Classification (1)	Supervised Learning (1)	ANN (1)	Kim (2024)
		Regression /forecasting (1)	Supervised Learning (1)	RF (1)	Olave-Rojas and Nickel (2021)
ABM&S-MC (1)	Predictive (1)	Classification (1)	Unsupervised Learning (1)	BN (1)	Shojaati and Osgood (2023)
DES-Markov (1)	Predictive (1)	Regression /forecasting (1)	Supervised Learning (1)	DT (1)	Nan et al. (2024)

#### 3.2.4 Programming language

A range of programming languages was used to develop the ML components within Hybrid M&S–ML studies. Of the 90 studies reviewed, 59 explicitly reported the programming language used, while the remaining 31 studies did not disclose this information or provided insufficient detail to infer the implementation language.

Python emerged as the most frequently used language, cited in 32 studies. Its popularity can be attributed to its flexibility, user-friendly syntax, and the availability of powerful ML libraries such as *Scikit-learn*, *TensorFlow*, and *Keras*. Python also integrates well with simulation libraries such as *SimPy*, making it a common choice in DES-related studies (e.g., Abuhayet al., 2023, Fairleyet al., 2019, Kovalchuket al., 2018).

R, mentioned in 12 studies, was primarily used for statistical modelling and data visualisation. *RStudio*, as a dedicated development environment, provided a strong platform for analytical tasks, although R was typically employed in conjunction with rather than fully embedded within simulation workflows (e.g., Hatnaet al., 2024, Kimet al., 2021, Ortiz-Barrioset al., 2024).

Java and MATLAB were each used in five studies. Java was most often associated with simulation platforms such as *AnyLogic*, which is Java-based, while MATLAB was used in more engineering-oriented studies, where its built-in support for mathematical modelling and control systems proved advantageous. In some cases, studies used multiple languages—Valtchev et al. (2021), for example, implemented ML components in Python and simulation models in AnyLogic, illustrating the modularity and loose coupling often found in hybrid systems.

Other languages included C (used in three studies) and Julia (in two studies), typically selected to meet specific computational or modelling needs, or due to researcher expertise (e.g., Wanget al., 2024, Cockrell and Axelrod, 2023).

**Fig. 7:**
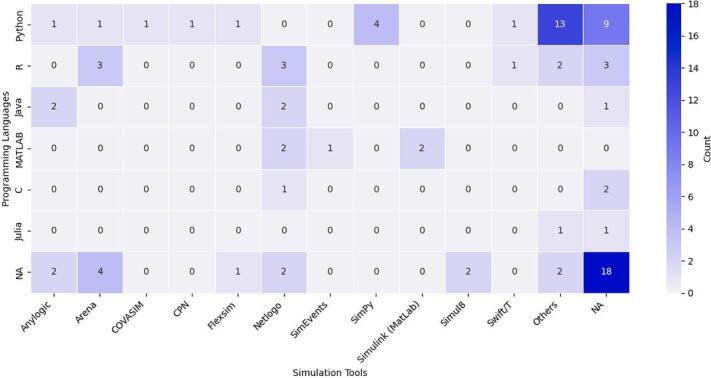
Programming Language by Simulation Tools in Hybrid M&S–ML Studies.

Fig. 7 presents the distribution of programming languages used in conjunction with simulation software. Studies in which the programming language was not reported or could not be determined were labelled as NA. It can be argued that the presence of multiple programming languages and tools across several reviewed studies underscores the modular and decoupled nature of many Hybrid M&S–ML implementations, particularly in sequential integration designs (see Section 3.2.5.4). In such cases, simulation and ML components are developed independently and linked through intermediate data exchanges, allowing researchers to select languages most appropriate for each modelling task.

#### 3.2.5 Integration of M&S with ML

##### 3.2.5.1 Roles of ML across the M&S process

**Fig. 8:**
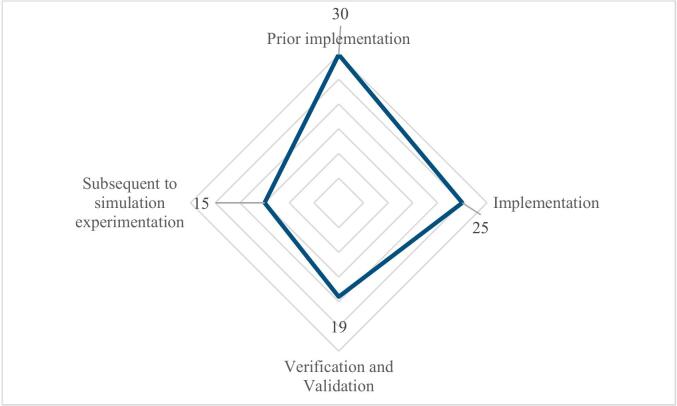
ML Roles Across the M&S Process in Hybrid M&S–ML Healthcare Studies.

Simulation studies are typically structured around key stages, including problem formulation, model development, verification and validation, experimentation, and output analysis (Brailsfordet al., 2019, Robinson, 2008). Building on this structure, we have analysed the integration of ML within M&S studies into four phases: pre-implementation, during implementation, during experimentation, and post-simulation (Fig. 8).

In the **pre-implementation** phase, 30 studies (34 %) applied ML to support model conceptualisation and calibration. The main functions identified were informing the structure of the simulation model (e.g., Dianovet al., 2022, Ortiz-Barrioset al., 2023, Sulis and Terna, 2021), initialising model parameters (e.g., Olave-Rojas & Nickel, 2021; Ortiz-Barrios et al., 2024; Tang, 2024), and enhancing data understanding through classification, preprocessing, or forecasting (e.g., Gradonet al., 2021, Kimet al., 2021, Valiet al., 2022). In this context, ML provided a means of embedding empirical regularities into the model prior to execution, reducing reliance on assumptions and improving alignment with observed system behaviour. For instance, Dianov et al. (2022) trained a fuzzy neural network on sociological survey data to extract behavioural decision rules, which were then encoded into an ABM&S to generate agent-level behaviour grounded in empirical evidence. Olave-Rojas and Nickel (2021) predicted ambulance travel speeds from operational covariates using ML, with these estimates supplying dynamic travel-time parameters to DES and thereby enhancing the realism of emergency medical service modelling. Similarly, Gradon et al. (2021) combined text-mining and sentiment analysis to identify misinformation sources, incorporating the outputs into an ABM&S of information diffusion to ensure that agent interactions reflected real-world communication patterns.

In the **implementation** phase, 25 studies (28 %) embedded ML directly within the simulation engine. The principal roles were parameter estimation (e.g., Alimet al., 2024, Cockrellet al., 2022, Nanet al., 2024), model emulation (e.g., Larieet al., 2021, Laskowskiet al., 2009, Wornowet al., 2023), and agent decision-making (e.g., Khatami and Gopalappa, 2021, Kim, 2024, Monostoriet al., 2021). Here, ML did not simply prepare the simulation but acted as a functional component of the modelling process itself, either by learning mappings between system inputs and outputs, approximating the behaviour of computationally intensive models, or supporting adaptive decision rules in dynamic environments. For instance, Alim et al. (2024) demonstrated this by training an ML model on a limited set of simulation runs to estimate key performance metrics such as patient waiting times and throughput. These ML-generated estimates were then passed to an optimisation module, enabling efficient exploration of policy alternatives without the need to rerun the full simulation. Larie et al. (2021) developed a workflow combining a mechanism-based ABM&S with ML to train predictive ANNs that serve as a surrogate model for the temporal dynamics of sepsis. By mapping short time-series sequences of simulated biomarkers to future health states, the surrogate reproduced the stochastic behaviour of the ABM&S at a fraction of the computational cost, thus supporting large-scale experimentation. Khatami and Gopalappa (2021) integrated reinforcement learning into a stochastic ABM&S of HIV progression, allowing the learning agent to adaptively identify testing and care-retention policies by interacting with the simulated population. This hybrid modelling design enabled the discovery of intervention strategies that balanced health outcomes and resource constraints while capturing system-level uncertainties.

During **experimentation**, 19 studies (21 %) employed ML to strengthen verification, validation, and sensitivity analysis (e.g., Abu Sufianet al., 2024, Anirudhet al., 2022, Cess and Finley, 2023). At this stage, ML was primarily used to generate auxiliary predictions, detect inconsistencies, or quantify uncertainty, thereby providing an additional layer of scrutiny to assess model robustness. The advantage of this integration lies in its ability to compare mechanistic simulation behaviour with data-driven approximations, revealing whether the simulation preserves plausibility across diverse scenarios. For example, Abu Sufian et al. (2024) combined ensemble XGBoost with an ABM&S of cardiovascular disease progression to cross-validate simulated trajectories against ML predictions. Their approach incorporated external clinical datasets for calibration, alongside systematic verification using Brier scores, calibration curves, and scenario-based sensitivity testing, ensuring both predictive accuracy and consistency with established disease mechanisms.

In the **post-simulation** phase, 15 studies (17 %) applied ML to analyse outputs, detect latent structure, and generate actionable insights (e.g., Asgaryet al., 2021, Forbus and Berleant, 2023, Snyder and Smucker, 2022, Snellmanet al., 2024, Uriarteet al., 2017, Lutz and Giabbanelli, 2022, Hatnaet al., 2024, Salibaet al., 2023, Chiuet al., 2024). Here, ML acted as a tool for interpreting the often complex, multi-variable outputs of simulation studies, enabling researchers to move beyond descriptive output summaries to uncover patterns and decision-relevant knowledge. Asgary et al. (2021) trained predictive models on more than 125,000 vaccination-clinic simulation runs to provide a fast surrogate for outcome evaluation, allowing policymakers to assess clinic configurations without repeating computationally intensive experiments. Chiu et al. (2024) used an ABM&S to generate synthetic hepatitis C transmission data and then applied survival analysis models to estimate infection risk, thereby linking simulated outputs to recruitment strategies for clinical trials. Across these examples, ML served as a bridge between simulation outputs and decision-making, enabling a more targeted, interpretable, and actionable use of complex simulation results.

One study—Siettos and Russo (2013)—was excluded from this analysis, as it is a review paper and does not report ML integration at any specific simulation stage.

##### 3.2.5.2 M&S-ML integration setup

This section examines how specific ML algorithms have been paired with different M&S methods across the reviewed studies, highlighting common integration setups and patterns of algorithm selection.

**ABM&S** was most frequently integrated with ANNs, reported in 13 studies (e.g., Batataet al., 2018, Chopraet al., 2023, Tang, 2024). In this context, ANNs were typically employed to approximate non-linear decision rules at the agent level, enabling more realistic behavioural modelling and capturing emergent system dynamics. BN and RL were also common, each appearing in six studies (e.g., Abdulkareemet al., 2019, Guoet al., 2022, Jorgensenet al., 2022, Khatami and Gopalappa, 2021). These were mainly applied to probabilistic reasoning under uncertainty and to support adaptive decision-making in dynamic environments. A smaller group of studies adopted evolutionary algorithms and KNN approaches (e.g., Mohammadzadeh and Safdari, 2015, Sulis and Terna, 2021, Whiteet al., 2022), highlighting ABM&S’s flexibility in accommodating diverse ML methods for classification, optimisation, and behavioural learning.

In **DES**-based studies, ANNs again dominated, featuring in nine studies, while random forests were the next most frequent choice with five studies. These algorithms were typically applied to predictive modelling tasks (e.g., Medvedet al., 2018, Timilehin and van Zyl, 2021) and to input calibration (e.g., Alimet al., 2024, Borestaet al., 2024). For example, Timilehin and van Zyl (2021) integrated an SEIR compartmental model with a DES, supported by a surrogate ANN optimised through a genetic algorithm, to generate fast and accurate predictions of COVID-19 dynamics and resource demand. This reduced computational burden while providing decision-makers with timely forecasts for managing lockdown strategies and hospital capacity. Similarly, Boresta et al. (2024) employed an ANN as a metamodel to approximate DES outputs in a multi-objective optimisation of ED resources. This approach enabled efficient calibration of parameters to minimise waiting times and operational costs, significantly accelerating the search for Pareto-optimal solutions. In DES applications, ANNs were generally used for demand forecasting and time-series approximation, while RFs provided robustness for calibration and scenario generation, particularly when working with noisy or high-dimensional input data (e.g., Ortiz-Barrioset al., 2023, Snyder and Smucker, 2022).

**SD** appeared in only one study, paired with MLP for a classification task (Liu & Pu, 2022). A small number of studies employed HS configurations—such as ABM&S–DES or ABM&S–MC—where ANN, RF, and BN were integrated to support behavioural modelling, input generation, or uncertainty quantification (e.g., Kim, 2024, Nanet al., 2024, Olave-Rojas and Nickel, 2021, Shojaati and Osgood, 2023).

**Fig. 9:**
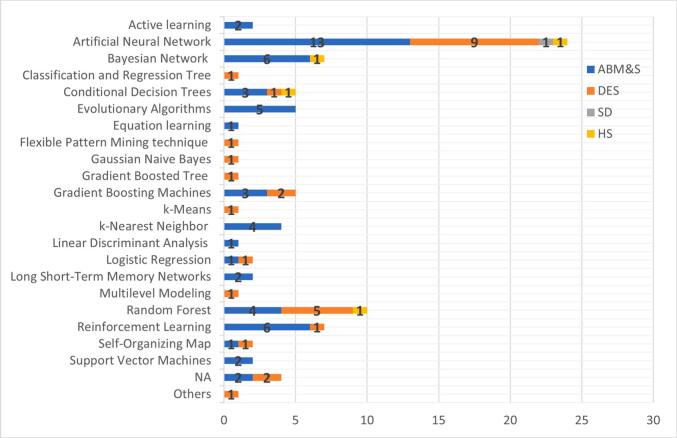
Distribution of ML Algorithms Across Simulation Modelling Techniques.

Taken together, these findings suggest that algorithm choice was not necessarily arbitrary, but often aligned with the simulation type and task requirements. ANNs were consistently favoured for non-linear approximation and surrogate modelling, RFs for calibration and forecasting under noisy conditions, and BN or RL for probabilistic or adaptive decision-making. Fig. 9 visualises these pairing patterns, while Table 4, Table 5, Table 6, Table 7 provide a detailed mapping of ML tasks, learning categories, and algorithm selection across simulation types.

##### 3.2.5.3 Data flow mechanism

This section examines how data exchange between M&S and ML components was managed across the reviewed studies, with a focus on the level of integration and interoperability mechanisms. Following the classification of Brailsford et al. (2019), three main approaches were observed: (i) manual exchange, (ii) intermediary-based integration, and (iii) direct implementation. These were further analysed in relation to the simulation software and programming languages used to uncover underlying technical patterns. Of the 90 studies reviewed, 57 provided sufficient detail to identify their data exchange mechanism. Table 9 presents a cross-tabulation of integration types by simulation software and programming language.

**Table 9:** Cross-analysis between data exchange and Software/programming language.

Simulation Software	ML Language	**Manual Data Exchange**	**Using an Intermediary Tool**	**Directly Implemented**	Not Available	Total
AnyLogic		2	3	–	–	5
	Java	1	1	–	–	2
	Python	–	1	–	–	1
	NA	1	1	–	–	2
Arena		5	1	–	2	8
	Python	1	–	–	–	1
	R	2	–	–	1	3
	NA	2	1	–	1	4
Covasim		–	–	–	1	1
	Python	–	–	–	1	1
CPN		–	–	–	1	1
	Python	–	–	–	1	1
Flexsim		2	–	–	1	2
	Python	1	–	–	1	1
	NA	1	–	–	1	1
NetLogo		2	2	1	5	10
	C	–	–	1	1	1
	Java	–	–	–	2	2
	MATLAB	1	–	–	–	2
	R	1	2	–	–	3
	NA	–	–	–	2	2
SimEvents		–	–	1	–	1
	MATLAB	–	–	1	–	1
SimPy		2	–	2	–	4
	Python	2	–	2	–	4
Simul8		1	–	1	–	2
	NA	1	–	1	–	2
Simulink		2	–	–	–	2
	MATLAB	2	–	–	–	2
Swift/T		1	–	–	1	2
	Python	–	–	–	1	1
	R	1	–	–	–	1
Others		6	3	1	8	18
	Julia	–	1	–	–	1
	Python	4	2	1	6	13
	R	2	–	–	–	2
	NA	–	–	–	2	2
NA		14	1	4	15	34
	C	1	1	–	–	2
	Java	1	–	–	–	1
	Julia	–	–	1	–	1
	Python	2	––	–	7	9
	R	2	––	1	–	3
		8	–	2	8	18
Total		37	10	10	33	90

**Manual exchange** was the most frequently reported, appearing in 37 studies. In these cases, M&S and ML models were developed independently, with their outputs exported in structured formats (e.g., CSV or Excel) and then imported into the companion model (e.g., Mohammadzadeh and Safdari, 2015, Nanet al., 2024). This “offline coupling” is reported where tools such as Arena, NetLogo, SimPy, or AnyLogic were involved. For example, Hatna et al. (2024) used Python-based ML models to estimate epidemiological parameters, which were then manually inserted into a NetLogo-based ABM&S to simulate Shigella transmission. Similarly, Abu Sufian et al. (2024) generated COVID-19 forecasts in Python, which were manually input into a SimPy simulation. These coupled workflows appear to be selected primarily for their flexibility or as a response to software incompatibilities. They frequently utilise structured data formats, which remain the dominant type across the 90 reviewed studies.

**Intermediary-based integration** was reported in 10 studies. Here, scripting environments or third-party connectors were used to automate the data pipeline between simulation and ML modules, creating a “semi-automated coupling”. For instance, Rabbani et al. (2018) employed Arena’s VBA scripting interface to integrate simulation results with an external ML module for post-simulation optimisation. In another example, Abdulkareem et al. (2019) linked a BN with a NetLogo ABM&S using R as an intermediary via the R NetLogo extension, enabling runtime data interaction between the two components. Compared to manual transfer, intermediary solutions appear to reduce the risk of transcription errors and improve reproducibility, although their efficiency often depends on the stability and performance of the middleware.

**Direct implementation** was also observed in 10 studies, where ML methods were embedded directly into the simulation codebase, typically within a common programming language, such as Python (e.g., Kovalchuket al., 2018, Fairleyet al., 2019, Wornowet al., 2023). This “tight coupling” enables runtime prediction, adaptive parameter updates, and continuous learning. For example, Wornow et al. (2023) integrated ML algorithms within the Python-based APLUS framework, allowing models to update dynamically as the simulation progressed. Such designs appear to minimise data transfer overhead and support more adaptive workflows, although they often seem to depend on shared data structures and language compatibility.

Patterns across tools and languages appear to reflect these integration choices (Table 9). R frequently appeared in intermediary-based configurations, especially when coupled with NetLogo. Python-based environments, such as SimPy, were often associated with direct implementation, whereas proprietary platforms like Arena and Flexsim tended to rely more on manual data exchange. Overall, tighter integration seems easier to achieve when M&S and ML components operate within a common programming environment.

##### 3.2.5.4 Integration method

Integration between M&S and ML components is a key consideration in designing hybrid modelling workflows. In the context of HS, Brailsford et al. (2019) outlined four integration modes—sequential, enriching, interaction, and full integration—to describe how multiple simulation paradigms (e.g. DES, SD, ABM&S) can be structurally combined. These classifications, however, assume that each method contributes equally and structurally to the overall simulation logic, which does not always apply in Hybrid M&S–ML.

Unlike HS models, where simulation methods often operate at a similar conceptual level, ML in Hybrid M&S–ML frequently serves a supporting role. It is typically applied for tasks such as classification, forecasting, parameter estimation, or optimisation—functions that enhance simulation input, post-process simulation output, or inform model behaviour. In most studies, ML is not embedded as a structural element of the simulation engine. However, a subset of studies adopts more integrated designs in which ML operates during simulation execution, supports adaptive behaviours, or functions as a surrogate model. These varying roles highlight a spectrum of integration depth that is not fully captured by the original HS typology.

To better capture the range of integration designs in Hybrid M&S–ML, this review adopted a tailored three-category typology: Sequential Integration, Enrichment Integration, and Direct Integration. Integration approaches were either explicitly reported or could be reasonably inferred in 85 of the 90 studies, based on the authors’ interpretation of the methodological descriptions provided.

**Sequential integration** was the most common approach, identified in 58 studies. In this setup, M&S and ML components are developed and executed separately, with data exchanged at the boundaries of each stage (e.g., Tang, 2024; Ortiz-Barrios et al., 2024; Vali et al., 2022). In this type of integration, ML is typically applied prior to simulation—to estimate parameters, generate input data, or initialise agent behaviour—or following simulation execution to support output analysis. A key limitation of this approach is the restricted feedback during runtime. However, it provides advantages in terms of transparency and modularity, as ML and simulation can be developed separately, facilitating replication and sensitivity testing (e.g., Borestaet al., 2024, Khatami and Gopalappa, 2021, Ortiz-Barrioset al., 2024). For example, Abdulkareem et al. (2018) employed a BN to guide agents’ perceptions of cholera risk in an ABM&S, while Ozik et al. (2018) used ML to construct a metamodel from simulation results, which then informed further experimental runs.

Sequential integration was mostly associated with predictive ML tasks, observed in approximately 62 % of such studies (e.g., Jorgensenet al., 2022, Valtchevet al., 2021, Atalan et al., 2022). For example, Jorgensen et al. (2022) used ML surrogate models to approximate stochastic agent-based simulations, enabling efficient Bayesian inference for tumour growth and infectious disease models. Similarly, Atalan et al. (2022) applied ensemble ML algorithms such as AdaBoost and Gradient Boosting to predict ED outputs, with forecasts subsequently integrated into a DES framework to evaluate healthcare resource costs and performance. In a related application, Valtchev et al. (2021) generated synthetic simulation data to train an AI model for assessing SARS-CoV-2 testing strategies in schools, where the predictive outputs informed the evaluation of intervention scenarios.

**Enrichment integration** was observed in 10 studies, where ML was used to enhance the design or interpretation of simulations without being embedded in the runtime. This form of integration included tasks such as model calibration, development of agent-level logic, or post-simulation analysis to improve interpretability. For instance, Cockrell et al. (2021) applied a nested active learning framework to refine clinically meaningful parameter ranges in a sepsis model. Snellman et al. (2024) used SOM and PCA to cluster and visualise the outputs of 100,000 simulations, enabling a more meaningful interpretation of results.

In Enrichment setups, ML was often used in predictive or prescriptive tasks, particularly where simulation calibration or latent structure estimation was required (e.g., Gartner and Padman, 2020, Hatnaet al., 2024, Sulis and Terna, 2021). For example, Gartner & Padman (2020) applied supervised classification methods to predict over-, correct, and underestimation of ED waiting times with 70–78 % accuracy, and then integrated these ML-derived attributes into a DES. This enrichment allowed testing how staffing decisions affect both actual and perceived waiting times, providing actionable insights for ED management. In Hatna et al. (2024), ML was used to rank intervention parameters based on their importance in reducing bacterial infection transmission, and these insights were incorporated into the simulation model design to refine calibration and scenario evaluation. Unlike sequential integration, where ML predictions serve primarily as static inputs to the simulation, enrichment integration integrates ML insights into the design and logic of the simulation, even if the ML component is not embedded at runtime.

**Direct integration** was observed in 17 studies, where ML was embedded within the simulation runtime to enable real-time interaction. This design supports adaptive behaviour and continuous learning, but it typically requires shared data structures and language compatibility. RL was especially prominent in this category, reported in four studies. For example, Cess and Finley (2023) integrated neural networks into an ABM&S to calibrate tumour growth simulations against image data dynamically, while Ying and Lin (2023) built the FAIOFS model in which fuzzy rules were continuously updated with sensor inputs. Such tight coupling enables prescriptive applications, including adaptive control and policy optimisation.

Direct integration was most frequently associated with prescriptive ML applications, including control systems and RL-driven decision-making (e.g., Abdulkareemet al., 2020, Hijry, 2024, Lazebnik, 2023, Shuvoet al., 2024). For example, Abdulkareem et al. (2020) embedded ML within an ABM&S to dynamically adjust individual and collective risk-coping strategies based on simulated social interactions. Similarly, Lazebnik (2023) integrated a deep RL agent into a hospital staff and resource allocation simulation to optimise patient treatment outcomes in real time, while Hijry (2024) combined IoT-collected data with a DES and ML control to reduce patient waiting times and enhance workflow efficiency in an ED. Unlike enrichment or sequential setups—where ML is mostly applied before or after simulation runs to inform model inputs or interpret outputs—these studies demonstrate direct, runtime integration, allowing ML to influence simulation behaviour adaptively as it executes.

### 3.3 Study outcome (O)

#### 3.3.1 Level of implementation

This section classifies the level of implementation reported in Hybrid M&S–ML studies in healthcare, using a three-stage framework adapted from Kar et al. (2024) and Brailsford et al. (2019): *Proof of Concept*, *Potential for Real-World Implementation*, and *Actual Real-World Implementation*. These categories reflect a continuum of model maturity, ranging from conceptual feasibility to practical deployment, providing insight into the extent to which hybrid approaches have progressed toward operational use.

**Proof of Concept** was assigned to 14 of the 85 studies where hybrid methods were introduced within controlled, theoretical, or synthetic contexts. These studies typically relied on limited or artificial datasets and focused primarily on demonstrating methodological feasibility or technical innovation, rather than practical application or validation. The emphasis in this group was on model architecture, integration design, or computational performance (e.g., Gehlotet al., 2022, Mesabbahet al., 2019, Stolfi and Castiglione, 2020).

**Potential for Real-World Implementation** encompassed 70 studies that addressed real-world healthcare challenges, including hospital operations and resource management (e.g., Alim et al., 2024; Ortiz-Barrios et al., 2024; Theeuwes et al., 2021), infectious disease modelling and public health interventions (e.g., Al-Baziet al., 2024, Hatnaet al., 2024, Salibaet al., 2023), clinical decision support and treatment pathways (e.g., Abu Sufianet al., 2024, Barneset al., 2020, Shojaati and Osgood, 2023), and biological or physiological modelling (e.g., Monostoriet al., 2021, Schneckenreitheret al., 2021, Tang, 2024). These studies frequently employed real-world or high-fidelity simulated data and were motivated by practical objectives. However, they generally fell short of actual deployment. Validation was commonly conducted through retrospective comparisons, scenario-based experiments, or expert feedback (e.g., Gartner and Padman, 2020, Hijry, 2024, Theeuwes et al., 2021).

Only two studies were categorised under **Actual Real-World Implementation**. These works described either system deployment or prospective field testing, including validation against empirical outcomes or real-time decision-support use cases. In addition to technical evaluation, these studies considered practical aspects of implementation, such as usability, stakeholder engagement, and integration into existing operational environments (e.g., Yousefi et al., 2018).

#### 3.3.2 Validation and verification

Validation and verification (V&V) are critical for establishing the credibility and reliability of Hybrid M&S–ML models, particularly in healthcare, where outputs may influence high-stakes or safety-sensitive decisions. Consistent with Kar et al. (2024) on hybrid simulation in healthcare, the reporting of V&V also remains limited across the Hybrid M&S–ML literature.

Among the 90 studies analysed, 37 explicitly reported validation activities. Of these, the majority were Type A (application-focused) studies (19 studies, e.g., Asgaryet al., 2021, Timilehin and van Zyl, 2021, Sulis and Terna, 2021), while 16 were classified as Type B (frameworks supported by application) (e.g., Chopraet al., 2023, Lazebnik, 2023, Uriarteet al., 2017). The most common validation strategy was comparison with real-world datasets, including retrospective analysis or benchmarking against empirical data (e.g., Jorgensenet al., 2022, Chenet al., 2019). However, in many cases, validation was only briefly mentioned, with limited information on the procedures followed, making it difficult to assess the rigour or scope of the evaluation.

Verification was reported in only three studies, all of which were Type A. These studies applied methods such as internal consistency checks (e.g., Snyder and Smucker, 2022, Schneckenreitheret al., 2021) and code or logic verification procedures (e.g., Perumal & van Zyl, 2020).

A total of 11 studies reported conducting both validation and verification, with an even split between Type A and Type B classifications (e.g., Alimet al., 2024, Yousefi and Yousefi, 2020). These studies tended to adopt more comprehensive evaluation strategies, including scenario-based testing (e.g., Ortiz-Barrios et al., 2024), iterative calibration (e.g., Ortiz-Barrioset al., 2024), and validation against empirical benchmarks or known system behaviours (e.g., Cockrell & Axelrod, 2023). Details from these studies suggest a more rigorous evaluation process, although the depth of reporting still varied.

**Fig. 10:**
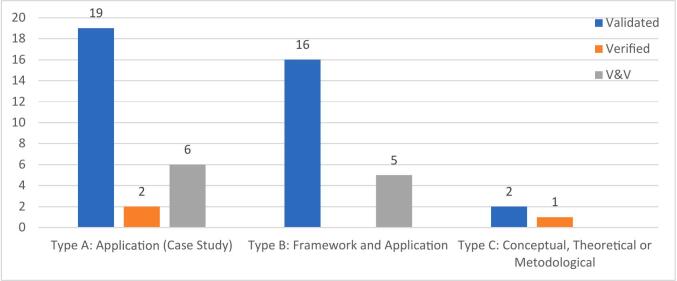
Validation and Verification Practices by Study Type.

Both studies classified under actual real-world implementation reported performing both validation and verification. However, this was not universally the case: several studies that reported both V&V activities remained at conceptual or pre-implementation stages. This suggests that while V&V can support implementation readiness, its presence alone does not guarantee model maturity or deployment outcomes. Fig. 10 provides a summary of validation and verification practices by study type, highlighting the variation in methodological thoroughness across different categories of hybrid studies.

## 4 Discussion

### 4.1 Addressing research questions

In this section, we revisit the research questions presented in Section 1 and reflect on key insights into the current state of Hybrid M&S–ML integration within healthcare applications. We discuss the primary application areas, themes, integration techniques, methodological trends, the synergy between M&S and ML, and the key motivations driving this integration.

*RQ1: What healthcare domains are most frequently targeted in hybrid M&S–ML studies, and how do these application areas relate to the nature of studies conducted?*

The review reveals increasing interest in Hybrid M&S–ML approaches in healthcare, with a marked acceleration after 2020—likely catalysed by the COVID-19 pandemic. The pandemic posed significant operational and policy challenges, prompting the development of simulation- and ML-based tools to support forecasting, resource management, and system-level resilience. This is reflected in the dataset, where 23 of the 64 post-2021 studies (36 %) explicitly addressed COVID-19-related applications, underscoring the relevance of hybrid approaches in navigating complex, uncertain healthcare environments.

The healthcare domains explored in the reviewed studies were categorised into four main themes, as outlined in Section 3. 1. While these studies varied in scope and methodological focus, they shared several common priorities—namely, the incorporation of real-time data, the development of advanced ML algorithms to improve system responsiveness and efficiency, and the enhancement of model adaptability through the integration of additional behavioural, clinical, or contextual variables.

In terms of study classification, the field remains strongly application-driven. Type A (application-based) and Type B (frameworks with illustrative applications) studies together accounted for 91 % of the reviewed literature. The most frequently targeted domain was hospital operations and resource management (37 studies), followed by infectious disease modelling and public health interventions (33 studies), and clinical decision support and treatment pathways (15 studies). These patterns suggest that hybrid approaches are primarily employed to address immediate, operational-level problems in healthcare, where the predictive and prescriptive capabilities of ML can directly enhance the representational power of M&S. The relative absence of Type C (theoretical or methodological) studies indicates a current lack of foundational work that critically examines the underlying assumptions, integration frameworks, or generalisability of Hybrid M&S–ML methods. This gap highlights an opportunity for future research to contribute conceptual clarity and methodological innovation to what remains a largely empirical and implementation-oriented field.

The methodological alignment of M&S techniques with application areas further reinforces these findings. DES was predominantly used in studies focused on hospital operations, reflecting its suitability for modelling workflows, queues, and resource allocation in structured environments. In contrast, ABM&S dominated the other three categories, particularly in applications requiring the modelling of heterogeneous agents, dynamic interactions, and behavioural adaptation—key characteristics in public health, clinical care, and epidemiological modelling.

*RQ2: Which M&S techniques, ML tasks, algorithms, and integration approaches are most prevalent in hybrid M&S–ML studies, and what patterns emerge in their application across different implementation levels?*

Our analysis reveals clear preferences in the integration of M&S techniques with specific software tools. ABM&S is the most used method, featured in 62 % of studies, and is predominantly developed using open-source platforms such as NetLogo and SimPy. In contrast, DES, appearing in 32 % of studies, is more frequently developed using commercial platforms such as Arena and AnyLogic. The data suggest that while open-source tools dominate in ABM&S studies, commercial tools are more prevalent in resource-intensive, operational simulations—reinforcing the tendency to select platforms aligned with specific modelling requirements.

SD was notably underrepresented, appearing in only one study (1 %) of the reviewed sample. This is unexpected given its historical prominence in healthcare simulation and its more visible role in earlier reviews of HS (e.g., Sallehet al., 2017, Karet al., 2024), where SD appeared alongside DES and ABM&S in a more balanced distribution. he scarcity of SD–ML integration in healthcare also contrasts with other fields, where ML and data science techniques have been applied more frequently for tasks such as parameter estimation, model optimisation, and risk assessment (e.g., Codyet al., 2019, Rodriguez-Ulloa, 2018, Xi-Zhe, 2013). Including SD in our scope was therefore deliberate: its limited presence signals a methodological gap rather than irrelevance, pointing to an underexplored but potentially valuable avenue for future research in healthcare contexts. In terms of M&S–ML combinations, as shown in Fig. 9, ABM&S is frequently paired with ANNs and BNs to support agent-level optimisation, classification, and decision-making. ABM&S is also commonly combined with EA and KNN for similar classification and optimisation tasks. In DES-based studies, ANNs are often used for forecasting and demand estimation, while RF, GBM, DT, and LR are applied for predictive modelling and input calibration.

Regarding ML programming language preferences, these choices often reflect non-technical and methodological constraints—such as researcher familiarity, library availability, institutional norms, or platform requirements—rather than integration-specific technical considerations. Our analysis highlights several practical factors shaping language use. Python is the most frequently used language, appearing in 35 % of studies, due to its flexibility, extensive ML libraries, and seamless integration with simulation environments such as SimPy. R, used in 13 % of studies, is primarily applied for statistical modelling and visualisation, often in combination with other simulation tools.

Language–platform alignment was clearly observed. Python was mostly used with open-source simulation environments such as SimPy and Swift/T. NetLogo, although primarily a simulation platform, was often extended using additional languages, including Java, R, and MATLAB, demonstrating its flexibility in hybrid configurations. Our findings also reveal a preference for integrating multiple languages within hybrid systems, suggesting a modular, loosely coupled design where simulation and ML components are developed independently and linked via data exchanges, offering flexibility in language selection and ensuring compatibility with diverse research needs.

*RQ3: What are the stated aims and functional roles of ML within hybrid M&S–ML models in healthcare studies?*

The analysis of ML tasks within hybrid M&S–ML frameworks (As detailed in Section 3.2.2) revealed key insights into the functional roles of ML across different simulation paradigms. Predictive analytics was the most prevalent application, appearing in 64 % of studies, with regression and forecasting emerging as the dominant approaches—particularly for predicting patient outcomes and system performance. Prescriptive tasks, present in 30 % of studies, were focused on optimisation and decision-making. These typically involved ML models such as reinforcement learning or optimisation algorithms to guide adaptive decisions, marking a significant advancement in supporting real-time healthcare management. By contrast, descriptive tasks—which aim to identify latent structures or patterns in data—were notably underrepresented, appearing in only 6 % of studies. Arguably, this underutilisation points to an opportunity for further research, particularly in the use of ML for exploratory analysis and the identification of emergent behaviours in complex, high-dimensional healthcare systems. In terms of learning categories, supervised learning dominated, appearing in 70 % of studies. Among the algorithms used, ANNs and RF were the most frequently employed.

*RQ4: How are ML methods deployed across the stages of the M&S lifecycle, and what integration setups and data flow mechanisms are used to facilitate this deployment?*

The analysis of healthcare studies revealed the contribution of ML across various M&S stages (3.2.3, 3.2.5.1). Findings show that in 34 % of studies, ML was applied during the pre-implementation stage, primarily supporting problem identification by detecting anomalies, inefficiencies, or emergent behaviours within healthcare systems. In 28 % of studies, ML facilitated implementation tasks, including parameter tuning and rule specification—automating processes that would otherwise be manually intensive. ML also enhanced input data handling, enabling more effective preprocessing, feature selection, and generation of realistic inputs—crucial for accurate simulation outcomes. In 21 % of studies, ML supported model verification, helping to ensure confidence in simulation results through comparison with empirical data or inferred behaviours. Additionally, during the post-simulation stage (reported in 17 % of studies), ML was used to support results analysis, employing techniques such as clustering and dimensionality reduction to interpret simulation outputs and uncover meaningful trends. These findings illustrate how ML contributes not only to analytical enhancement but also to addressing practical modelling challenges across the simulation lifecycle. A key trend emerging from this analysis is the gradual decline in ML usage across later M&S stages. This may be attributed to two factors: (1) the increasing maturity and reliability of simulation models and tools, which reduce the need for further data-driven refinement, and (2) a more developed understanding of input–output relationships, which lessens the perceived necessity for ML support in later stages.

In terms of integration techniques, the review identifies three main configurations (Section 3.2.5.4). Sequential integration is the most prevalent, observed in 58 studies (68 %), where ML is applied either before or after the simulation—for instance, to predict input variables or interpret outputs. This approach is most associated with predictive tasks and improves model precision and operational efficiency. Enrichment integration, identified in 10 studies (13 %), involves the use of ML to calibrate models or refine post-simulation analysis without runtime interaction. It is typically associated with predictive and prescriptive tasks, such as model tuning or stakeholder-oriented interpretation. Direct integration, reported in 17 studies (20 %), embeds ML within the simulation runtime itself. This configuration supports real-time adaptation, particularly in prescriptive contexts, with reinforcement learning commonly used to facilitate dynamic decision-making. These findings suggest that while ML is still primarily used as an external analytic layer, there is growing interest in more interactive and embedded architectures, especially in studies requiring real-time responsiveness.

The review also examined data flow mechanisms between ML and M&S components (Section 3.2.5.3). Manual data exchange remains the most common method, found in 37 studies, where structured file formats such as CSV or Excel are used to transfer data between independently developed components. This approach is typically associated with platforms like Arena, NetLogo, and SimPy and is well-suited to loosely coupled systems, particularly where software incompatibilities exist. Integration via intermediary tools, reported in 10 studies, facilitates more dynamic communication using scripting environments or APIs—for example, Arena’s VBA scripting or R extensions for NetLogo. These setups allow runtime interaction and help reduce human error. Direct integration, also found in 10 studies, involves tight coupling between ML and simulation, usually within a shared programming environment. This allows for real-time responsiveness and is particularly relevant to applications involving decision support or adaptive behaviours. A key observation is that software–language pairings strongly influence integration choices. For example, SimPy–Python combinations were often used for direct integration due to their inherent compatibility (e.g., Fairleyet al., 2019, Kovalchuket al., 2018). Conversely, tools like NetLogo, which support extensions in Java, R, and MATLAB, were employed across all three integration types, offering greater flexibility for hybrid implementations (e.g., Abdulkareemet al., 2018, Al-Baziet al., 2024, Yousefi and Yousefi, 2020). Notably, only two studies explicitly reported the data format used, although structured formats were implicitly assumed in the majority of cases.

The adapted typology described in 3.2.5.3, 3.2.5.4 provides a more appropriate lens through which to analyse the structure and depth of integration in hybrid M&S–ML studies. Sequential integration was observed consistently across all stages of the simulation lifecycle (e.g., Cockrell and Axelrod, 2023, Khatami and Gopalappa, 2021, Mesabbahet al., 2019), reflecting its flexibility and compatibility with diverse study designs. Enrichment integration was most frequently applied in the pre-implementation and post-simulation stages (e.g., Chopraet al., 2023, Gartner and Padman, 2020, Kimet al., 2021), while direct integration was concentrated during implementation (e.g., Lazebnik, 2023, Nanet al., 2024, Perumal and van Zyl, 2020), where adaptive control and runtime decision-making were required.

*RQ5: How are hybrid M&S–ML models in healthcare validated, verified, and implemented across different study types and maturity levels?*

The analysis indicates that although the majority of studies (70; 82 %) extend beyond conceptual development and engage with real-world healthcare challenges, a clear gap remains between model design and actual implementation. The Proof-of-Concept stage, represented by 14 studies (16 %), continues to focus on feasibility and integration design rather than deployment. In contrast, only two studies (2 %) reached the Actual Real-World Implementation stage, highlighting the ongoing difficulty of translating hybrid M&S–ML models into operational settings.

This trend is consistent with earlier findings by Brailsford et al. (2019), who observed that only 2 % of hybrid simulation studies achieved real-world implementation. A similar pattern has been reported in the broader M&S literature in healthcare, where deployment rates remain low—typically around 5 %—as noted by Brailsford et al. (2009) and Katsaliaki and Mustafee (2011). These findings suggest that despite methodological advances and increased attention to practical healthcare applications, implementation in operational environments remains limited.

V&V practices were also found to be inconsistently applied. While some studies offered validation through empirical comparison, scenario analysis, or expert input, detailed reporting was often lacking. Verification was rarely addressed explicitly. This lack of clarity undermines the transparency and reproducibility of the models, particularly in high-stakes healthcare contexts where robust evaluation is essential.

### 4.2 Future research directions

Based on the findings, four research priorities are identified to address current limitations and guide the advancement of Hybrid M&S–ML integration in healthcare:

i- Real-Time and Dynamic Data Integration

A key direction for future research is to incorporate real-time data streams into hybrid models, enabling more adaptive and context-aware simulations. Real-time data can enhance model responsiveness and predictive performance. Several studies (e.g., Asgaryet al., 2021, Hoet al., 2023, Oziket al., 2021) have highlighted the value of dynamic data in enhancing the operational relevance of simulation models. Future work should focus on developing algorithms and infrastructures capable of handling high-frequency data in real-world healthcare settings.

ii- Advancements in GenAI and Computational Techniques.

The integration of Generative AI (GenAI) and large language models with M&S and ML holds considerable promise for transforming healthcare applications. GenAI can automate the processing of unstructured data—such as medical images and patient records—into structured formats suitable for simulations. This would enhance decision-making in areas like patient flow optimisation and resource allocation. Additionally, GenAI can automate feature engineering, generate synthetic data, and improve model robustness, particularly in data-scarce scenarios. Our review did not identify any studies that combined GenAI with traditional M&S in healthcare within the review period. However, recent research has begun to explore this integration, particularly through LLMs, by enabling the extraction, reconstruction, and critique of simulation model structures from textual descriptions (Akhavan and Jalali, 2024, Giabbanelli et al., 2025, Monks et al., 2025, Yanget al., 2025). These developments suggest that integrating GenAI into M&S presents diverse opportunities for advancement and should be explored in future research.

iii- Automation of Data Handling and Integration

Managing large, diverse datasets remains a major challenge in Hybrid M&S–ML healthcare applications. Despite the potential benefits, most studies still rely on manual data integration, which is time-consuming and prone to error. Only 12 % of studies utilised automated data exchange, a critical limitation for real-time decision-making. Future research should focus on developing automated systems for data exchange that span the entire data lifecycle—covering collection, preprocessing, feature extraction, and validation. Advanced techniques, such as Natural Language Processing (NLP) for textual data and computer vision for medical imaging, could further enhance data integration and improve the predictive capabilities of healthcare simulations.

iv- Development of a Standardised Framework for Hybrid M&S–ML in Healthcare.

To improve consistency and reproducibility in Hybrid M&S–ML studies, the development of a standardised framework is essential. Von Rueden et al. (2020) have outlined a domain-independent framework that categorises integration subfields and methodological approaches. Building on this foundation, the need in healthcare is for a framework that is more application-focused, guiding integration across stages such as data collection, preprocessing, model development, and validation. Such a framework should also take into account the challenges particular to healthcare, including data heterogeneity, interdisciplinary working, and the translation of models into practice. Addressing these considerations would enhance transparency, reduce methodological inconsistencies, and increase the credibility and adoption of Hybrid M&S–ML in healthcare.

These insights offer a comprehensive understanding of Hybrid M&S–ML integration in healthcare, laying the groundwork for future research and practical applications. By addressing existing challenges and capitalising on emerging opportunities, the integration of M&S and ML has the potential to significantly advance healthcare decision-making and operational efficiency.

## 5 Conclusion

In recent years, M&S and ML have become central to addressing complex challenges, optimising processes, and supporting data-driven decision-making in healthcare. The increasing integration of ML with traditional M&S methodologies highlights the complementary strengths of these disciplines in tackling data-intensive problems. This review systematically examined 90 relevant studies, focusing on the methodological and technical aspects of Hybrid M&S–ML in healthcare applications.

The findings were organised around the rationale for hybridisation, the simulation and ML techniques employed, integration methods, programming languages and software tools, and the role of ML across simulation stages. We also examined the synergy between M&S and ML in healthcare applications, alongside levels of implementation and the state of validation and verification practices. The results show that ML is particularly effective in supporting the generation of insights from complex datasets, thereby enhancing the capabilities of simulation models. However, several gaps persist in integration design, verification and validation reporting, and real-world deployment.

This review offers a structured and integrated perspective on Hybrid M&S–ML applications in healthcare, providing a foundation for both academic inquiry and applied innovation. A limitation is that the analysis was restricted to the Web of Science database, which is considered one of the most comprehensive sources of peer-reviewed research, but may not capture all relevant studies. Future reviews could be strengthened by incorporating additional databases such as Scopus or PubMed, alongside citation-chasing approaches, to ensure broader coverage.

## Funding statement

This work was partially funded by the National Institute for Health and Care Research (NIHR) Applied Research Collaboration Kent, Surrey, Sussex. The views expressed are those of the authors and not necessarily those of the NHS, the NIHR or the Department of Health and Social Care.

## CRediT authorship contribution statement

**Ali Ahmadi:** Writing – original draft, Software, Methodology, Data curation, Investigation. **Masoud Fakhimi:** Writing – review & editing, Validation, Supervision, Methodology, Conceptualization, Formal analysis. **Carin Magnusson:** Writing – review & editing, Validation, Supervision.

## Declaration of competing interest

The authors declare that they have no known competing financial interests or personal relationships that could have appeared to influence the work reported in this paper.

## Data Availability

No data was used for the research described in the article.
